# Fairness Evaluations of Higher Education Graduates’ Earnings: The Role of Female Preference for Equality and Self‐Interest

**DOI:** 10.1111/1468-4446.13192

**Published:** 2025-01-29

**Authors:** Anna Zamberlan, Diana Roxana Galos, Susanne Strauß, Thomas Hinz

**Affiliations:** ^1^ Department of Sociology LMU Munich Munich Germany; ^2^ Department of Sociology University of Copenhagen Copenhagen K Denmark; ^3^ Center for the Experimental‐Philosophical Study of Discrimination Aarhus University Aarhus Denmark; ^4^ Department of History and Sociology University of Konstanz Konstanz Germany

**Keywords:** earnings gap, fairness evaluations, gender, higher education, perceptions of inequality, survey experiment

## Abstract

Educational and occupational horizontal segregation contribute significantly to economic inequalities, especially in contexts with a strong correspondence between fields of study and occupational outputs, such as in Germany. However, the extent to which individuals perceive disparities in economic returns across different fields of study as fair and the factors influencing these fairness evaluations remain largely unexplored. This study aims to understand fairness evaluations by assessing two theoretical explanations and their interrelation: (1) female preference for equality, where women generally favour smaller earnings disparities, and (2) biases leading to higher reward expectations for individuals in the same field of study as the evaluator. Our empirical research draws on a novel survey experiment from the German Student Survey (2021), in which higher education students evaluated the fairness of realistic earnings for graduates from various fields of study. These earnings relate to the entry phase of an individual's career, reflecting differences in economic returns exclusively tied to fields of study, independent of occupational or life trajectories. Our findings support the female preference for equality and self‐interest theoretical perspectives, revealing that women and respondents in fields associated with lower‐earning jobs tend to perceive greater unfairness. We further find evidence of an interaction between the two mechanisms, with women being particularly likely to perceive greater unfairness when it aligns with their self‐interest.

## 1 Introduction

Understanding perceptions of fairness in earnings is highly relevant from both distributive justice and individual perspectives. Perceiving economic rewards as unfairly low can lead to various adverse effects, including increased stress levels (Murtaza et al. 2021), decreased job performance (Jones and Skarlicki 2003), and reduced life satisfaction (Adriaans 2022). Disparities in labour market outcomes, such as earnings, are closely connected to individuals' fields of study and occupational segregation (Charles and Grusky 2005). Enrolment in different fields of study influences the range of occupations one can successfully apply for and, therefore, earnings. In particular, economic returns to education vary by the gender composition of fields of study. Female‐dominated subjects tend to lead to occupations with lower wages on average compared to gender‐neutral and especially male‐dominated ones (Leuze and Strauß 2014; Levanon, England, and Allison 2009). Several factors have been used to explain the existence and persistence of lower earnings in predominantly female occupations. These include the devaluation of female fields and professions (England 1999, 2018), the lower degree of skill specificity in such occupations (Goldin 2014; Klein 2011), and status beliefs that consider men and male‐dominated fields or occupations as being more valuable than women and female‐dominated fields or occupations (Cuddy, Fiske, and Glick 2008; Ellemers 2018).

It remains less clear, however, whether fairness evaluations of a given distribution of economic returns are judged differently based on the characteristics and current situation of those making the judgements. In other words, it remains an open question whether justice principles are universal or if respondent characteristics can shed light on the mechanisms underlying fairness evaluations. Some studies focussing on the evaluation of others' earnings in specific contexts suggested that both men and women consider lower wages for women to be fair (e.g. Auspurg, Hinz, and Sauer 2017 for Germany; Jasso and Webster 1997 for the city of Baltimore). The fact that women, the social category disadvantaged in terms of earnings, tend to accept their lower earnings has been discussed as the ‘contented female worker’ phenomenon. However, more recent studies based on data from different European countries and focussing on individuals' perceptions of their own earnings have shown that women are actually more likely to perceive their earnings as too low (Adriaans and Targa 2023), especially if they work in occupations with a higher proportion of women (Brüggemann and Hinz 2023). The variation in findings across studies may be attributed to differences in their designs, as well as to potential changes over time in the awareness of gender pay inequalities and perceptions of their fairness. In summary, respondent gender remains a relevant yet insufficiently understood factor in shaping evaluations of earnings fairness. This underscores the importance of further investigating the role of gender and the potential mechanisms underpinning gender differences in fairness evaluations. Existing contributions have also highlighted the salience of individuals' identification with the social situation being assessed, suggesting that individuals are likely to base their justice evaluations on self‐interest (Deutsch 1975; Van Lange et al. 2013). Therefore, in the context of fairness evaluations of the economic returns to fields of study, an individual's field of study may shape their evaluations.

Our study aims to contribute to the understanding of fairness evaluations and the underlying channels by focussing on the characteristics of individuals judging the fairness of earnings, particularly their gender and self‐identification with the social situation being evaluated. We consider one key setting associated with the stratification of economic rewards: *fields of study*. To our knowledge, this is the first study to scrutinise whether individuals justify labour market disparities based on different educational specialisations. From an analytical point of view, this focus has two major advantages. First, given the significance of social comparisons in justice evaluations (Jasso, Törnblom, and Sabbagh 2016) and the different labour market returns to various fields of study (Altonji, Blom, and Meghir 2012; Jacob and Weiss 2010; Klein 2011, 2016), earnings disparities shaped by an individual's choice of field of study represent a suitable context for examining fairness evaluations. Additionally, as the earnings associated with the different fields of study evaluated by respondents relate to the entry phase of an individual's career (more details are provided in the research design and analytic strategy section), they reflect differences in economic returns to fields of study at labour market entry, unaffected by individuals' subsequent careers. This is a relevant advantage over existing studies where the earnings being evaluated often reflect occupational and family formation factors (as they are respondents' own earnings or mainly refer to a later stage in the career) that are not under the researchers' direct control.

We conducted our study in Germany, which is characterised by a strong correspondence between fields of study and occupational outcomes (Jacob and Weiss 2010; Klein 2011, 2016; Leuze 2007). This makes Germany a particularly interesting setting for investigating fairness evaluations of economic returns related to fields of study. We draw on a novel survey experiment administered through the German Student Survey, a representative survey of students in German higher education conducted in 2021. The questionnaire included an original survey experiment on perceptions of the fairness of economic returns for graduates in different fields of study. Specifically, each student was asked to evaluate the fairness of the earnings differential between graduates from two different (randomised) disciplines. This setting enables meaningful comparisons between five large fields of study and complements previous studies on fairness evaluations based on respondents' earnings (e.g. Adriaans and Targa 2023; Brüggemann and Hinz 2023).

### 1.1 Perceptions of Fair Pay: Why Gender and Fields of Study Matter

Perceptions of fairness regarding the different economic returns to fields of study are likely to reflect shared stereotypes about these fields and related occupations, their value, and, therefore, the appropriate financial reward. Gender is among the most studied and debated factors shaping such stereotypes. According to the devaluation perspective, gender biases lead to valuing female‐dominated jobs less than male‐dominated jobs, even if these jobs are of comparable worth (England 1999, 2018). Similarly, status characteristics theory argues that gender stereotypes embody status elements, where men, as a social group, tend to be advantaged compared to women (Ridgeway 2011). In most societies, these status beliefs associate men and anything considered ‘male’ with a higher level of competence and worthiness than women and anything considered ‘female’ (Cuddy, Fiske, and Glick 2008; Ellemers 2018).

Given these premises, it might generally be expected that gender stereotypes influence perceptions of the fairness of earnings obtained by graduates in fields of study characterised by different gender compositions. If male‐dominated fields of study are assigned a higher perceived status, they are also associated with stereotypes that encompass higher economic rewards compared to female‐dominated fields of study (Cohen and Huffman 2003; Kilbourne et al. 1994). In line with this prediction, some single‐country studies show a general acceptance of lower wages for women as fair. This is evident both in assessments of the fairness of others' earnings (see experimental evidence by Auspurg, Hinz, and Sauer 2017; Sauer 2020) and in evaluations of individuals' own earnings (based on observational data by Pfeifer and Stephan 2019; Valet 2018). Most interestingly for our contribution, the acceptance of lower wages for women holds regardless of respondents' characteristics, including gender. This suggests that gender stereotypes not only exist and influence fairness evaluations but are also widely shared, even by those whom they disadvantage.

Yet, other theoretical perspectives point to a relevant role of the characteristics of individuals expressing their fairness perceptions. In this contribution, we aim to go beyond the existing literature pointing to shared stereotypes favouring male fields and disfavouring female ones by testing two perspectives rarely considered in sociological research on the fairness of earnings: female preference for equality and self‐interest.

#### 1.1.1 Female Preference for Equality

The first reason it is reasonable to expect that fairness evaluations differ depending on individuals' characteristics is the existence of gender differences in justice principles. Previous studies have highlighted that social preferences, such as inequality aversion, are gendered, with women being more socially oriented and having a greater tendency to respond to unfairness than men (for an overview of studies in economics, see Eckel and Grossman 2008).

Recent sociological studies, while not explicitly testing the female preference for equality thesis, have found pronounced gender differences in fairness evaluations. In particular, women are more likely than men to perceive their own earnings as unfair (Adriaans and Targa 2023; Brüggemann and Hinz 2023). Brüggemann and Hinz (2023) examined the ‘contented female worker’ paradox in 27 European countries using data from the European Social Survey, finding that women are less satisfied with their pay than men. This finding is supported by Adriaans and Targa (2023), who illustrated using the same data that women in 15 out of 28 European countries reported stronger perceptions of unfairness regarding their earnings. Given that women's earnings are generally lower than men's, this finding may reflect that women are indeed ‘discontented’ with (gender) earnings inequalities. In other words, women may be characterised by justice principles that emphasise equality more than men in the evaluation and distribution of resources (Beutel and Marini 1995). However, since these studies rely on respondents' assessments of their own earnings, it is not possible to disentangle women's preference for equality from self‐interest. Indeed, women's higher perceptions of unfairness regarding their earnings (which are typically lower than men's) could stem from either of these mechanisms.

The argument that women prefer equality is further supported by experimental evidence in the field of distributive justice, such as in ‘dictator games’, where individuals make decisions about how to distribute resources between themselves and other recipients (for an overview of these experimental studies, see Croson and Gneezy 2009; Eckel and Grossman 2008). Assessments of others' earnings or dictator games are better suited to disentangling the different mechanisms underlying fairness evaluations. Empirical evidence shows consistent gender disparities in behaviour, with women typically exhibiting more generosity and concern for fairness, while men tend to prioritise their own output (Andreoni and Vesterlund 2001; Doñate‐Buendía, García‐Gallego, and Petrović 2022, for a recent meta‐analysis of 136 experiments involving dictator games).

We aim to contribute to the sociological literature on fairness evaluations by estimating the difference in assessments of the fairness of economic returns to fields of study between male and female respondents. In line with the female preference for equality perspective, our first hypothesis states.

Hypothesis 1 *Women are more likely than men to evaluate earnings differentials related to fields of study as unfair.*

#### 1.1.2 Self‐Interest

The second reason why respondent characteristics might influence fairness perceptions is that individuals identify with elements of the social situation being evaluated, making their self‐interest a salient mechanism (Deutsch 1975; for a review, see Van Lange et al. 2013). This is especially the case in situations with an imbalance in resource distribution (Wade‐Benzoni, Tenbrunsel, and Bazerman 1996). In line with this perspective, empirical studies have illustrated that self‐interest is a dominant principle on which individuals rely when assessing justice evaluations (Cook and Hegtvedt 1983; Deutsch 1975; Rodriguez‐Lara and Moreno‐Garrido 2012).

When applied to fairness evaluations of earnings differentials between graduates from different fields of study, respondents may identify with one element of the comparison based on their own field of specialisation. Thus, when assessing the fairness of earnings for graduates from specific fields of study, they may be likely to consider higher earnings for fields similar to their own as fair and lower earnings as unfair. We can thus formulate two complementary hypotheses based on the self‐interest principle.

Hypothesis 2a *Earnings gaps in favour of a field of study are perceived as fair by students enrolled in the same subject compared to students enrolled in different subjects.*

Hypothesis 2b *Earnings gaps that disfavour a field of study are perceived as unfair by students enrolled in the same subject compared to students enrolled in different subjects.*

#### 1.1.3 The Interrelation Between Female Preference for Equality and Self‐Interest

The two theoretical perspectives explored so far focus on gender and the (mis)match between the respondent's field of study and the field being evaluated to explain the mechanisms underlying fairness evaluations. However, gender and field of study are closely interrelated factors. Earnings disparities are closely linked to educational and occupational segregation (Charles and Grusky 2005). Specifically, fields of study with differing gender compositions are associated with varying economic returns once individuals enter the labor market: female‐dominated fields tend to lead to lower‐paid jobs compared to gender‐neutral or male‐dominated fields (Leuze and Strauß 2014; Levanon, England, and Allison 2009).

Given the gender segregation in fields of study, the lower levels of perceived unfairness among male students and the higher levels among female students may reflect not only a female preference for equality but also self‐interest, as men are more likely to dominate higher‐paying fields, while women tend to dominate lower‐paying ones. Similarly, self‐interest mechanisms may be confounded with the effect of student gender, as female students, who are often enrolled in fields with lower economic returns, tend to favour equality more than male students, who dominate higher‐paying fields. While the gender composition of fields of study can be accounted for statistically, its role in shaping perceptions of unfairness warrants explicit exploration.

One might also expect a significant interaction between the two mechanisms. If both female preference for equality and self‐interest are present, then female students enrolled in fields of study with lower economic prospects are the group most likely to perceive unfairness. Conversely, male students enrolled in fields of study with higher economic returns are the least likely to perceive earnings differentials as unfair. To test the relationship between these two mechanisms, we estimate the interaction between students' gender and their match or mismatch with the field of study being evaluated. Accordingly, our third set of hypotheses is as follows.

Hypothesis 3a *Women are more likely than men to evaluate earnings differentials between fields of study as unfair, particularly when they are enrolled in the same field of study with low economic prospects as the one in the comparison.*

Hypothesis 3b *Men are less likely than women to evaluate earnings differentials between fields of study as unfair, particularly when they are enrolled in the same field of study with high economic prospects as the one in the comparison.*

To facilitate an overview of our theoretical expectations, Table 1 summarises the perspectives and hypotheses tested in our study.

**TABLE 1:** Overview of the theoretical hypotheses and the underlying rationale.

	Hypotheses	Argument
Female preference for equality	Women are more likely than men to evaluate earnings differentials related to fields of study as unfair (Hypothesis 1).	Perceptions of fairness are gendered, with women being more likely to prioritise a balanced distribution of economic resources compared to men.
Self‐interest	Earnings gaps in favour of a field of study are perceived as fair by students enrolled in the same subject compared to students enrolled in different subjects (Hypothesis 2a).	Fairness evaluations are based on the relative positioning of one's own field of study within the distribution of economic resources: Those who benefit from this distribution are more likely to perceive it as fair, while those who are penalised by it are more likely to perceive it as unfair.
	Earnings gaps that disfavour a field of study are perceived as unfair by students enrolled in the same subject compared to students enrolled in different subjects (Hypothesis 2b).	
Interrelation between female preference for equality and self‐interest	Women are more likely than men to evaluate earnings differentials between fields of study as unfair, particularly when they are enrolled in the same field of study with low economic prospects as the one in the comparison (Hypothesis 3a).	If both female preference for equality and self‐interest are at play, there is a significant interaction between students' gender and the relative position of their field of study within the distribution of economic resources.
	Men are less likely than women to evaluate earnings differentials between fields of study as unfair, particularly when they are enrolled in the same field of study with high economic prospects as the one in the comparison (Hypothesis 3b).	

### 1.2 Research Design and Analytic Strategy

#### 1.2.1 Research Design

To evaluate these different theoretical perspectives empirically, we relied on the 2021 German Student Survey (Becker et al., 2024a, 2024b), an extensive representative survey of students in German higher education, including both universities and universities of applied sciences. The survey collected information from students enrolled in German tertiary education institutions on various topics, including their sociodemographic characteristics, educational path, and perceptions and attitudes towards political and societal issues. After excluding cases with missing data in the variables of interest, the analytic sample included 15,759 students. Supporting Information S1: Table S1 in the supplementary material presents descriptive statistics and details on missing cases.

For our analysis, we used a survey experiment added to the thematic module on inequality and fairness evaluations. Each respondent was asked to assess pay differentials in yearly earnings for labour market entrants after graduation in two randomly assigned fields of study, using a five‐point Likert scale ranging from ‘not at all justified’ to ‘fully justified’. Each respondent rated one pay differential, making the experimental design a between‐subject design. The motivation underlying this survey experiment was based on previous experimental studies in the German context that highlighted the salience of information on earnings related to different educational paths for inequality perceptions (Lergetporer and Woessmann 2021; Lergetporer, Werner, and Woessmann 2021). The possible comparisons involved five of the most common subject groups for students in higher education in Germany, namely humanities (€ 35,000), economics (€ 43,000), law (€ 46,500), engineering (€ 47,500), and medicine (€ 53,000). The earnings associated with each subject represented the average gross yearly earnings for individuals who graduate in these subjects in Germany (values were taken from the salary report for graduates by Hermann, Pela, and Stanski 2018/19). This approach provided all respondents, including those with little or no work experience, with the same realistic information about actual earnings in the German labour market. Moreover, it allowed us to test assessments of fairness related to the actual system of distribution of economic rewards after graduation.

It is important to note that, in the experiment, fields of study covaried with earnings, as the latter were realistic and thus inevitably correlated with specific fields. This interdependence made it challenging to disentangle the effects of these two dimensions and may raise questions about the specific relevance of fields of study, which is a key focus of this paper. However, our robustness checks provided reassurance that qualitative information about fields of study offered more insight into (un)fairness mechanisms than numerical data on the size of the earnings gap (see Robustness checks section).

#### 1.2.2 Analytic Strategy

In the first analytic step, we presented descriptive statistics on fairness evaluations based on respondents' gender and by the (mis)match between respondents' field of enrolment and the field shown in the comparison. Regarding the latter measure, we constructed a variable to distinguish between combinations where there was a mismatch (i.e. none of the fields shown in the experiment matched the respondent's field), where the respondent was enrolled in the same higher‐paid field shown in the comparison, and where the respondent was enrolled in the same lower‐paid field shown in the comparison.

In the second step, we conducted linear probability models to evaluate the earnings differential. To facilitate the interpretation of the results and to focus consistently on perceptions of unfairness, we transformed the original five‐point Likert scale into a dichotomous one: unfairness (including the original categories 5 ‘not at all justified’ and 4) versus indifference/fairness (including categories 3, 2, and 1 ‘fully justified’). Our substantive results did not vary when we relied on the original scale of the dependent variable. The main explanatory variables are the respondents' gender and the (mis)match between their field of enrolment and the fields shown in the experiment. Relying on these variables allowed us to test the two theoretical perspectives of interest in a straightforward way. We presented the results in the form of predicted probabilities to provide a more informative account of the levels of perceived unfairness. However, we systematically referred to significance tests related to regression coefficients (presented in the supplementary material) to test the hypotheses appropriately.

We tested the *female preference for equality hypothesis* by examining whether male and female respondents differ in their overall fairness evaluations, net of respondents' field of study. The hypothesis is supported if the respondent's gender significantly influences fairness evaluations, with women being more prone to perceive earning differentials as unfair.

We tested the *self‐interest thesis* by analysing whether respondents enrolled in the same higher‐paid field shown in the experimental comparison (match, higher‐paid field) are less likely to perceive unfairness toward the earnings differential than respondents enrolled in different fields, net of their gender. Additionally, we examined whether respondents enrolled in the same lower‐paid field shown in the experimental comparison (match, lower‐paid field) are more likely to perceive unfairness than those enrolled in different fields.

Finally, we tested the *interrelation between female preference for equality and self‐interest* by including the interaction between gender and field (mis)match in our models. This approach helped to disentangle the two mechanisms.

Due to the randomised experimental design, respondents' characteristics are orthogonal to the comparison between the fields of study they were asked to evaluate. However, their characteristics (including gender and field of enrolment) were not experimentally manipulated. Consequently, their relationship with the outcome variable might be biased. We controlled for individual characteristics likely to confound the primary relationships of interest (which is particularly relevant when the explanatory variable is field of study (mis)match) or mediate it (which is more relevant when the explanatory variable is respondent gender). We included controls for age, migration background, university type (whether a student is enrolled at a university or a university of applied sciences), and federal state. Furthermore, we applied weights accounting for the sampling strategy and population characteristics. The first weight adjusted for the probability of inclusion, while the second was a normalised weight that adjusted for official statistics related to federal state, sex, field of study, type of tertiary education institution, age, and migration background (for more details, see Kroher et al. 2023). To account for the nesting of students in different higher education institutions, we clustered standard errors at the university level. Descriptive statistics for all variables included in the models are presented in Supporting Information S1: Table S1.

## 2 Results

### 2.1 Descriptive Statistics

**FIGURE 1:**
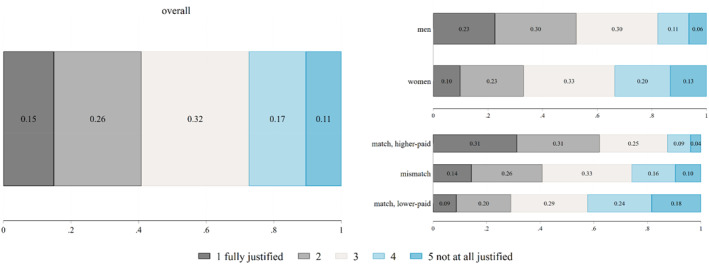
Fairness evaluations of earnings differentials overall and by students' characteristics; proportions. N: 15,759. *Source:* German Student Survey (Die Studierendenbefragung in Deutschland), 2021.

Figure 1 shows descriptive statistics about fairness evaluations overall (left panel) and based on respondents' attributes (right panels). Most respondents view the wage differential as fair: 41% selected the first two categories on the fairness scale, indicating a justified gap, while only 28% chose the last two categories, indicating a non‐justified gap. However, notable differences emerge when considering respondents' personal characteristics.

Focussing on gender, men are more likely to perceive the earnings differential as fair or to be indifferent: 83% of men reported the gap as fully justified or justified or chose the central category of the scale, compared to 66% of women (the difference between men's and women's responses is statistically significant: *p*‐value < 0.001). Conversely, women are more likely to perceive the differential as unfair: 33% considered the gap unjustified or not at all justified, compared to 17% of men (*p*‐value for the difference < 0.001).

Considering students' field of study and their match or mismatch with the fields shown in the experiment, those enrolled in the same field of study as the higher‐paid one shown are less likely to find the earnings gap unfair, with only 13% choosing categories 4 and 5 on the scale, indicating a non‐justified gap. This proportion increases to 26% for students enrolled in a different field from those shown in the experiment (with a statistically significant difference between the categories ‘match, higher‐paid’ and ‘mismatch’: *p*‐value < 0.001). The percentage rises to 42% for respondents enrolled in the same field of study as the lower‐paid one shown in the comparison (with a statistically significant difference compared to the ‘mismatch’ category: *p*‐value < 0.001).

To provide a more nuanced descriptive picture, Table 2 presents fairness evaluations based on the specific comparison between fields of study provided to respondents. Relatively high proportions of perceived unfairness are evident for comparisons involving humanities as the lower‐paid field, particularly when compared with economics (22% of respondents consider the earnings differential not at all justified). In contrast, comparisons involving medicine as the higher‐paid subject tend to be perceived as relatively fair. The highest percentage of respondents who deemed the earnings gap as fully justified is observed when medicine is compared with economics (28%).

**TABLE 2:** Fairness evaluations of earnings differentials through comparisons of fields of study; proportions.

Fields of study comparison	1	2	3	4	5	*N*
	Fully justified				Not at all justified	
Humanities versus economics	0.10	0.17	0.25	0.26	0.22	1582
Humanities versus law	0.09	0.20	0.27	0.26	0.18	1549
Humanities versus medicine	0.19	0.33	0.27	0.16	0.05	1680
Engineering versus humanities	0.12	0.24	0.23	0.23	0.18	1561
Engineering versus economics	0.17	0.23	0.37	0.14	0.09	1556
Engineering versus law	0.10	0.20	0.46	0.15	0.09	1575
Engineering versus medicine	0.16	0.30	0.34	0.14	0.06	1540
Law versus economics	0.09	0.22	0.43	0.15	0.11	1557
Medicine versus economics	0.28	0.37	0.24	0.08	0.03	1557
Medicine versus law	0.19	0.32	0.32	0.11	0.06	1602

*Note:* Unweighted proportions.

*Source:* German Student Survey (Die Studierendenbefragung in Deutschland), 2021.

### 2.2 Understanding (un)fairness Evaluations

**FIGURE 2:**
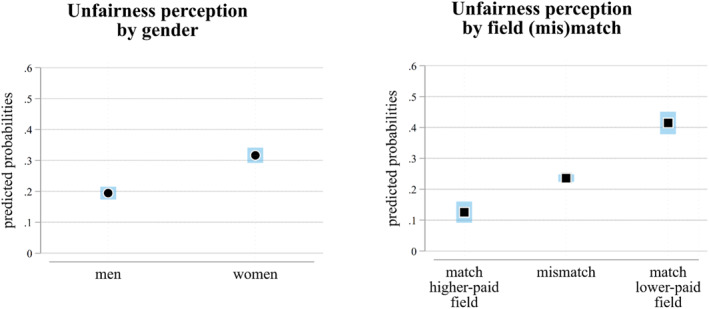
Predicted probabilities of perceiving unfairness by respondents' gender (left panel) and the match or mismatch between respondents' and experimental field of study (right panel) (95% c.i.). N: 15,759. Regression results in Supporting Information S1: Table S2 (Model 1). *Source:* German Student Survey (Die Studierendenbefragung in Deutschland), 2021.

Next, we turn to the interpretation of results from the linear probability models predicting perceptions of unfairness (using the dichotomous version of the dependent variable). These models include respondents' gender and the (mis)match of their field of study with the fields shown in the experiment as the main explanatory variables. Figure 2 illustrates the predicted probabilities of unfairness perceptions, presented by respondents' gender (left panel) and by field (mis)match (right panel).

As predicted by the female preference for equality scenario (H1) and as observed in the descriptive statistics in Figure 1, women exhibit significantly higher levels of perceived unfairness than men. The predicted probability of unfairness for women is slightly above 30%, while for men, it is around 20%.

Moreover, consistent with the self‐interest perspective (H2a and H2b), students enrolled in the same higher‐paid field shown in the experimental comparison (match, higher‐paid field) are the least likely to perceive unfairness, with a predicted probability slightly above 10%. In contrast, students enrolled in the same lower‐paid field shown in the comparison (match, lower‐paid field) are the most likely to perceive unfairness, with a probability of just over 40%. Respondents enrolled in a different field of study than those shown in the experiment (mismatch) have a predicted probability of about 25% of perceiving the comparison as unfair. These findings further corroborate the descriptive results presented in Figure 1.

Interestingly, these findings hold even when examining comparisons between specific fields of study (Supporting Information S1: Table S3 in the supplementary material). However, an exception arises when medicine is the higher‐paid field, as the statistical significance of respondents' gender and field (mis)match diminishes. This point will be further elaborated in the discussion section.

The previous findings underscore the importance of the female preference for equality and self‐interest perspectives in understanding perceptions of unfairness regarding earnings differentials among graduates across various fields of study. However, if both the female preference for equality and self‐interest perspectives are indeed influencing these perceptions, a significant interaction between these two mechanisms could be expected (H3a and H3b).

**FIGURE 3:**
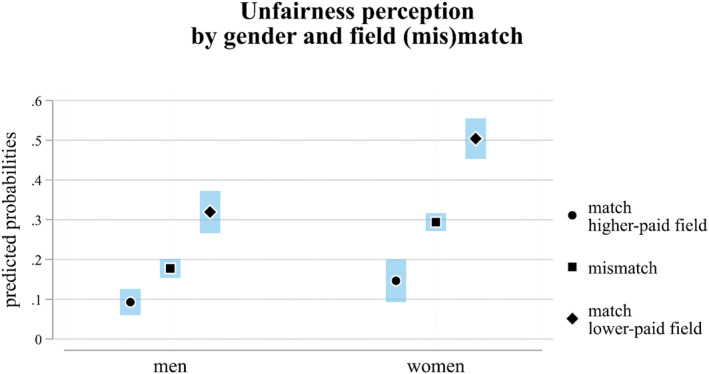
Predicted probabilities of perceiving unfairness by respondents' gender and the match or mismatch between respondents' and experimental field of study (95% c.i.). N: 15,759. Regression results in Supporting Information S1: Table S2 (Model 2). *Source:* German Student Survey (Die Studierendenbefragung in Deutschland), 2021.

Figure 3 presents results from a model that includes the interaction between gender and field (mis)match. In line with the self‐interest perspective, students enrolled in the same higher‐paid field as the one shown in the experimental comparison are the least likely to perceive unfairness. This trend is observed for both male (approximately 10%) and female (around 15%) students, with no statistically significant gender difference (*p*‐value = 0.0574). The lack of a significant gender difference for students enrolled in fields with high economic returns leads us to reject H3b. Respondents enrolled in a different field from those shown in the experiment have intermediate levels of perceived unfairness, with predicted probabilities of less than 20% for men and around 30% for women (and a statistically significant gender difference, see Supporting Information S1: Table S2, Model 2). Finally, students enrolled in the same lower‐paid field as the one in the comparison are most likely to perceive unfairness, with probabilities just over 30% for men and around 50% for women. This gender difference is both statistically significant (Supporting Information S1: Table S2, Model 2) and substantially relevant, supporting H3a.

In sum, women are consistently more likely than men to perceive unfairness across all categories of field (mis)match. However, the gender difference is especially pronounced among students enrolled in the same lower‐paid field as shown in the experimental comparison, indicating a significant interaction between the two mechanisms under investigation.

In summary, our results provide empirical support for the female preference for equality thesis and the self‐interest scenario. Women and students penalised by the structure of economic rewards for different fields of study (i.e. enrolled in lower‐paid fields) are significantly more likely to assess earnings differentials between graduates in different fields of study as unfair. Conversely, men and students who benefit from economic returns to their study subjects, namely those enrolled in higher‐paid fields, are less likely to perceive these earnings differentials as unfair. We further observe evidence of an interaction between the two mechanisms, in particular with women enrolled in fields with lower economic returns being the most likely to perceive differences in earnings returns between fields as unfair.

### 2.3 Robustness Checks

To test the robustness of our findings against our analytic choices, we explored the relevance of the numeric earnings information included in the survey. Given the perfect covariance between fields of study and realistic earnings, it could be argued that our results might reflect aspects other than the fields of study on which we base our interpretation, primarily the earnings level and especially the size of the earnings gap. We tested this by incorporating into the regression model a measure that captures the numeric size of the earnings differential (e.g. the comparison between engineering (€ 47,500) and humanities (€ 35,000) has an annual earnings gap of € 12,500). Specifically, we interacted the size of the earnings gap with both respondent gender and field (mis)match. The results of this robustness check are presented as predicted probabilities of unfairness perceptions by gender and gap size, as well as by field (mis)match and gap size in Supporting Information S1: Figure S1. They suggest that the relationship between the earnings gap size and unfairness perceptions is not well approximated by a linear trend. Specifically, medium values of the numeric gap are more likely to be perceived as unfair than wider and smaller earnings gap sizes.

It is relevant to note that the lowest levels of unfairness perceptions are observed in comparisons involving medicine as the higher‐paid field (Table 2), and additional analyses indicate lower and often not statistically significant effects of respondents' gender and field of study in fairness evaluations of earnings comparisons involving medicine (Supporting Information S1: Table S3). These results highlight the importance of qualitative information on the fields of study rather than numerical data on the size of the earnings gap. Therefore, with the main aim of testing the two theoretical perspectives of female preference for equality and self‐interest, in the primary analyses we focus on qualitative information about the respondents' field of study and the fields included in the comparison rather than on quantitative information about earnings and the size of the earnings differentials.

We further tested the robustness of our findings to the categorisation of combinations of respondent and experimental field of study as matches or mismatches. Specifically, we constructed a more nuanced measure of mismatch distinguishing between (1) mismatch where the respondent is enrolled in a higher‐paid field (i.e. one of the top two subjects: medicine or engineering); (2) mismatch where the respondent is enrolled in a lower‐paid field (i.e. one of the bottom two subjects: humanities or economics); and (3) pure mismatch, where the respondent is enrolled in law—situated in the middle of the earnings distribution—and evaluates a comparison excluding law, or in any case where the their field is absent from the experimental variations.

Supporting Information S1: Figures S2 and S3 show the results for the self‐interest scenario and the interaction between women's preference for equality and self‐interest, respectively. Although the predicted probabilities of unfairness are slightly higher for cases of pure mismatch compared to the other mismatch categories, in both graphs no significant differences are observed between different types of mismatch. This finding confirms that the interpretation of our main findings as self‐interest is correct, as it is the respondents' self‐identification with the situation being evaluated that matters, and not solely their affiliation to a highly‐ or poorly‐rewarded field of study.

## 3 Discussion

Whether we view it from a distributive justice perspective or an individual one, understanding perceptions of fairness in earnings is highly relevant (Adriaans 2022; Jones and Skarlicki 2003; Murtaza et al. 2021). Previous studies have highlighted the importance of fairness evaluations for the persistence of the gender wage gap, emphasising the role of an individual's gender in these perceptions, albeit with mixed results (e.g. Auspurg, Hinz, and Sauer 2017; Adriaans and Targa 2023). While gender appears to be a salient (yet unclear) determinant of fairness evaluations, it remains uncertain whether assessments of a given distribution of economic returns are judged differently according to the characteristics and current situation of those judging it.

Drawing on a novel survey experiment, we complement existing studies by focussing on economic disparities related to one of the primary drivers of financial returns in the labour market, namely the field of study, and by examining two potential channels contributing to individuals' fairness evaluations. Our contribution to the literature is therefore twofold.

First, to the best of our knowledge, this is the first study to analyse individuals' perceptions of the (un)fairness of different economic returns to fields of study. Choices of specific fields of study in higher education significantly determine the occupations available to graduates and, consequently, their earnings. This is particularly evident in the German context, which is characterised by a comparatively strong correspondence between fields of study and occupational trajectories (Jacob and Weiss 2010; Klein 2011, 2016; Leuze 2007). Utilising an original survey experiment that provides information on earnings at career entry, we ensure that fairness evaluations of earnings differentials are based solely on fields of study and do not reflect individuals' occupational or life trajectories.

Second, we test two different theoretical explanations underlying justice evaluations: (1) female preference for equality, whereby women prioritise a balanced distribution of economic resources compared to men, and (2) self‐interest, leading evaluators to justify higher earnings for graduates in fields of study similar to their own. We also test for the interaction between these two mechanisms. We find robust evidence supporting the thesis of female preference for equality (Hypothesis 1), as female respondents are more likely to perceive unfairness compared to men. This finding aligns with previous literature that shows that women are more likely to respond to instances of unfairness (Adriaans and Targa 2023; Brüggemann and Hinz 2023; Eckel and Grossman 2008; Strauß, Brügggemann, and Lang 2024) and, in contrast with other studies (Auspurg, Hinz, and Sauer 2017), it indicates that women are ‘discontented’ when it comes to earnings differentials. The female preference for equality explanation is complemented by the self‐interest one (Hypotheses 2a and 2b), as students tend to consider the higher (lower) earnings of graduates from the same field of study as their own as fair (unfair). In other words, an imbalance in resource distribution likely triggers self‐interest (Wade‐Benzoni, Tenbrunsel, and Bazerman 1996), leading students who benefit from the stratification system of economic rewards among fields of study to assess earnings differentials as fair compared to those who do not profit from it. Interestingly, the two mechanisms interact, such that the self‐interest leading students in lower‐paid fields to judge earnings differentials that penalise their own field as unfair is particularly pronounced for women (Hypothesis 3a).

In addition to shedding light on the mechanisms explaining fairness evaluations of earnings related to fields of study, our findings challenge the idea of a general devaluation of female fields and occupations (Cuddy, Fiske, and Glick 2008; Ellemers 2018; England 1999, 2018). In fact, female students and those enrolled in fields with comparatively low economic returns in the labour market, especially female students in lower‐paid fields, are particularly prone to perceive earnings differences related to different fields of specialisation as unfair. These differences typically involve male‐dominated fields being highly rewarded and female‐dominated fields being poorly rewarded.

While speculative, the lack of support for the gender devaluation perspective might partly reflect the characteristics of a sample comprising a young cohort of students enrolled in higher education institutions. Young and highly educated individuals might exhibit weaker gender stereotypes when evaluating fields of study and their economic returns. Our findings could also mirror a cohort change toward more gender equality, at least in terms of gender stereotypes regarding fields of study. Recent empirical evidence seems to support the thesis of a change in women's fairness evaluations towards greater awareness of unfair wages (Adriaans and Targa 2023; Brüggemann and Hinz 2023), particularly among younger cohorts (Strauß, Brügggemann, and Lang 2024). However, based on our cross‐sectional data, we cannot test whether less traditional gender stereotypes are characteristic of younger ages (across all birth cohorts) or more recent cohorts (throughout their entire life course).

Our study was conducted in Germany, where the choice of a specific field of study is highly consequential for later labour market returns, and it raises the question of the extent to which our findings are generalisable to different contexts. Replicating our study in countries with a weaker link between fields of study and students' labour market outcomes, such as the United Kingdom, might reveal different reactions to perceived unfairness among students. For example, in such countries, students might perceive earnings‐related unfairness to be more pronounced than in countries where the link between fields of study and occupations is more direct. In these latter contexts, students may already make strategic choices regarding their field of study, leading to a perception of fairness related to earnings imbalances.

Another aspect that may be worth exploring in more depth in future studies is the explicit motivations of respondents for judging a given comparison to be fair or unfair. In the data available to us, the respondents were asked to choose from a predefined list of 11 reasons (related to effort and responsibility, skill and economic relevance, and societal value) why they believed one field was better paid than the other (see section D in the supplementary material for details). Our exploratory analyses (Supporting Information S1: Figure S4) suggest that there are different reasons given by respondents for why one field of study is better paid than another, depending on the specific fields being assessed. While effort, responsibility, and social value are commonly invoked as explanations for the higher earnings for fields such as law and medicine, the higher earnings for engineering and economics are justified by the skills in demand in the labour market and their higher economic value. This finding may suggest that self‐interest is parallelled by a more articulate justification for the existing structure of financial rewards. In particular, respondents point to the labour market and the economic system itself—rather than effort or the social benefits deriving from specialisation in a given discipline—to justify higher earnings for a subject such as male‐dominated engineering as fair.

Even though students were presented with a generous list of reasons for their assessment of why one field should be better paid than another, they might have interpreted these reasons in various ways or had additional justifications. Future research could incorporate quantitative (e.g. open‐ended survey questions) and qualitative (e.g. interviews or focus groups) designs to explore the invoked justifications for earnings differentials.

Finally, some significant limitations should be acknowledged. First, the classification of fields of study in the survey experiment includes broad groups with varying numbers of subfields. More specific fields of study might have triggered different reactions in terms of (un)fairness perceptions. For example, it is possible that respondents' fairness evaluations would have differed with specific fields of study, such as psychology and philosophy, which are implicitly included under the broader category of ‘humanities’. These two more specific fields might be perceived as exhibiting different career opportunities, labour market demand, and societal value—factors that could influence respondents' perceptions of unfairness related to earnings.

Second, our experimental design involves covariation between fields of study and related earnings. By providing respondents with information on the real economic returns to different fields of study, we assessed fairness judgements and the underlying mechanisms related to the real labour market and its stratification of economic rewards that higher education students will face after graduation. This design feature is critical as it increases the external validity of our findings. At the same time, the covariation between fields and earnings makes it impossible to test the salience of these two measures independently, as would be possible if they varied orthogonally. While the information provided by the current design is suitable for testing the theoretical perspectives addressed in this article, it does not allow us to definitively determine whether students' judgements of fairness are driven by the level of earnings, the magnitude of earnings differentials, the specific fields of study compared, or characteristics of those fields, such as their gender composition. Future research on the topic might consider designing experiments with orthogonal variation between different theoretically relevant factors.

Understanding students' fairness evaluations of the returns to different fields of study is essential for comprehending the persistence of wage disparities in contemporary labor markets. This study aimed to explore fairness evaluations by testing two mechanisms, both of which received empirical support. We further found evidence in support of their interrelation. Specifically, our findings suggest that both female preference for equality and self‐interest shape fairness evaluations of higher education graduates' earnings. Moreover, these mechanisms interact, with women enrolled in lower‐rewarded fields of study being particularly prone to perceive earnings differentials between fields as unfair. These results mark a significant step toward addressing pay equity across different fields of study and occupations, as individuals who feel ‘discontented’ with the current system of economic rewards—often disadvantaging women and female‐dominated fields—could be motivated to respond to perceived unfairness.

## Conflicts of Interest

The authors declare no conflicts of interest.

## Supporting information

Supporting Information S1

## Data Availability

The data that support the findings of this study are available in both a downloadable, anonymised format and an on‐site‐only format. The latter option provides access to the most granular information, which is the same information we rely on in this study. More details are available on the data provider's webpage: https://metadata.fdz.dzhw.eu/en/data‐sets/dat‐sid2021‐ds1?page=1&size=10&type=surveys&version=1.0.1.
