# Supplementary material for: Fairness Evaluations of Higher Education Graduates’ Earnings: The Role of Female Preference for Equality and Self‐Interest
Source: Br J Sociol. 2025 Jan 29;76(3):541–52. doi: 10.1111/1468-4446.13192 (PMC12163563; doi:10.1111/1468-4446.13192)
Supplement: Supplementary file 1 — Supporting Information S1 [file BJOS-76-541-s001.docx]

Fairness evaluations of higher education graduates’ earnings:

the role of female preference for equality and self-interest

**Supplementary material**

**Section A: Survey experiment**

Further information in English and German can be found in the documents related to the dataset ‘The Student Survey in Germany (2021)’ at the following link: <https://metadata.fdz.dzhw.eu/en/data-sets/dat-sid2021-ds1?page=1&size=10&type=surveys&version=1.0.1>

**Original version in German**

E4_6 Gehaltsunterschiede zwischen Fächergruppen:

“Im Folgenden geht es um Gehaltsunterschiede zwischen Absolvent*innen verschiedener Fächergruppen und darum, ob Sie diese Unterschiede als gerechtfertigt empfinden. Absolvent*innen *Wirtschaftswissenschaftlicher* Fächer verdienen nach ihrem Abschluss in Vollzeit durchschnittlich etwa *43.000 €* pro Jahr (Bruttojahresgehalt). Absolvent*innen der *Geisteswissenschaften* verdienen demgegenüber durchschnittlich ca. *35.000 €* im Jahr.

In welchem Ausmaß finden Sie es gerechtfertigt, dass Absolvent*innen *Wirtschaftswissenschaftlicher* Fächer direkt nach ihrem Abschluss mehr verdienen als Absolvent*innen der *Geisteswissenschaften*?”

gar nicht gerechtfertigt völlig gerechtfertigt

**
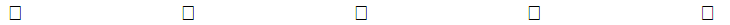
**

Anmerkung: Bei Frage E4_6 handelt es sich um ein Experiment in dem verschiedene Fächergruppen miteinander verglichen werden sollen. Dargestellt ist einer von zehn Fächervergleichen als Beispiel. Jede*r Befragte bekommt nur einen Fächervergleich angezeigt. Die in diesem Beispiel kursiv markierten Wörter zeigen, an welcher Stelle Informationen variiert werden. Die Varianten sind:

1. Wirtschaftswissenschaften (43.000 €) vs. Geisteswissenschaften (35.000 €)

2. Ingenieurwissenschaften (47.500 €) vs. Geisteswissenschaften (35.000 €)

3. Medizin (53.000 €) vs. Geisteswissenschaften (35.000 €)

4. Rechtswissenschaften (46.500 €) vs. Geisteswissenschaften (35.000 €)

5. Medizin (53.000 €) vs. Rechtswissenschaften (46.500 €)

6. Medizin (53.000 €) vs. Ingenieurwissenschaften (47.500 €)

7. Medizin (53.000 €) vs. Wirtschaftswissenschaften (43.000 €)

8. Rechtswissenschaften (46.500 €) vs. Wirtschaftswissenschaften (43.000 €)

9. Ingenieurwissenschaften (47.500 €) vs. Rechtswissenschaften (46.500 €)

10. Ingenieurwissenschaften (47.500 €) vs. Wirtschaftswissenschaften (43.000 €)

E4_7 Was könnte Ihrer Meinung nach den höheren Lohn in diesem Beispiel rechtfertigen?

Die Absolvent*innen der *Wirtschaftswissenschaften* verdienen mehr als Absolvent*innen der *Geisteswissenschaften*, weil sie…^1^

- eine wichtige gesellschaftliche Funktion übernehmen.
- hohe finanzielle Erträge erwirtschaften.
- eine längere Wochenarbeitszeit haben.
- ein höheres Anforderungsniveau im Studium hatten.
- eine Tätigkeit ausüben, die von größerer Bedeutung für die Volkswirtschaft ist.
- zur Sicherung der Lebensqualität in der Gesellschaft beitragen.
- mehr Verantwortung im Beruf übernehmen.
- über am Arbeitsmarkt stärker nachgefragte Kenntnisse und Fertigkeiten verfügen.
- komplexere Aufgaben im Beruf ausführen.
- geringe zeitliche/räumliche Flexibilität in ihrer Tätigkeit haben.
- mehr Stress/Belastungen am Arbeitsplatz haben.
- nichts davon

^1^Anmerkung: Auch hier werden wieder die kursiv gedruckten Wörter/Informationen variiert, je nachdem, welchen Vergleich der/die Befragte zuvor in Frage E4_6 gesehen hat.

**English translation**

E4_6 Assessment of salary differences:

“The following section deals with salary differences between graduates in different areas of study and whether you feel that these differences are justified. Graduates in *economics* earn on average about *€ 43,000* a year (gross annual full-time salary) when they enter the job market. By contrast, graduates in *humanities* earn on average about *€ 35,000* a year.

To what extent do you think it is justified that graduates in *economics* earn more than graduates in *humanities* right after graduation?”

not at all justified fully justified

**
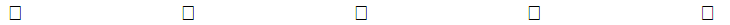
**

Note: Question E4_6 is an experiment in which different subject groups are to be compared with each other. As an example, one of ten subject comparisons is shown. Each respondent is only shown one comparison of subjects. The words marked in italics in this example show where information is varied. The variants are:

1. economics (€ 43,000) vs. humanities (€ 35,000)

2. engineering (€ 47,500) vs. humanities (€ 35,000)

3. medicine (€ 53,000) vs. humanities (€ 35,000)

4. law (€ 46,500) vs. humanities (€ 35,000)

5. medicine (€ 53,000) vs. law (€ 46,500)

6. medicine (€ 53,000) vs. engineering (€ 47,500)

7. medicine (€ 53,000) vs. economics (€ 43,000)

8. law (€ 46,500) vs. economics (€ 43,000)

9. engineering (€ 47,500) vs. law (€ 46,500)

10. engineering (€ 47,500) vs. economics (€ 43,000)

E4_7 In your opinion, what could justify the higher salary in this example?

Graduates in economics earn more than graduates in the humanities as they...^1^

- fulfil an important social function.
- generate high financial returns.
- have a longer working week.
- had a higher level of requirements in their studies.
- have a job that is of greater importance to the national economy.
- contribute to securing the quality of life in society.
- take on more responsibility at work.
- have knowledge and skills that are more in demand in the labour market.
- perform more complex tasks at work.
- have less temporal/spatial flexibility in their work.
- have more stress/strain at work.
- none of the above

^1^Note: Again, the words/information in italics are varied depending on the comparison the respondent previously saw in question E4_6.

**Section B: Descriptive statistics and regression tables**

**Table S1.** Descriptive statistics.

|  | **mean/ proportion** | **std. dev.** | **min-max** | **N** | **% missing** (from the initial sample of students who received the survey experiment) |
| --- | --- | --- | --- | --- | --- |
|  |  |  |  |  |  |
| Sex |  |  |  |  | 1.49% |
| Men | 0.40 |  |  | 6,299 |  |
| Women | 0.60 |  |  | 9,460 |  |
| Field of study (mis)match |  |  |  |  | 1.37% |
| Match, higher-paid field | 9.06 |  |  | 1,427 |  |
| Mismatch | 74.59 |  |  | 11,755 |  |
| Match, lower-paid field | 16.35 |  |  | 2,577 |  |
| Age | 24.16 | 4.66 | 18–49 | 15,759 | 1.85% |
| Place of birth |  |  |  |  | 0.09% |
| Germany | 0.91 |  |  | 14,344 |  |
| Abroad | 0.09 |  |  | 1,415 |  |
| Type of university |  |  |  |  | 0% |
| University | 0.62 |  |  | 9,701 |  |
| University of applied science | 0.38 |  |  | 6,058 |  |
| State |  |  |  |  | 0% |
| BB | 0.02 |  |  | 257 |  |
| BE | 0.04 |  |  | 589 |  |
| BW | 0.14 |  |  | 2,255 |  |
| BY | 0.13 |  |  | 2,115 |  |
| HB | 0.01 |  |  | 125 |  |
| HE | 0.07 |  |  | 1,147 |  |
| HH | 0.02 |  |  | 323 |  |
| MV | 0.02 |  |  | 309 |  |
| NI | 0.10 |  |  | 1,598 |  |
| NW | 0.29 |  |  | 4,627 |  |
| RP | 0.03 |  |  | 509 |  |
| SA | 0.03 |  |  | 452 |  |
| SH | 0.02 |  |  | 290 |  |
| SL | 0.02 |  |  | 242 |  |
| SN | 0.03 |  |  | 536 |  |
| TH | 0.03 |  |  | 385 |  |
| Weight (design) | 1.00 | 0.61 | 0.60–8.71 | 15,759 | 0% |
| Weight (redressment) | 0.98 | 0.87 | 0.27–14.90 | 15,759 | 3.28% |

Source: German Student Survey (Die Studierendenbefragung in Deutschland), 2021.

**Table S2.** Linear probability models of unfairness perception.

|  | **Model 1:**  **Female preference for equality & Self-interest** | **Model 2:**  **Female preference for equality*Self-interest** |
| --- | --- | --- |
|  | Coefficient (robust std. err.) | Coefficient (robust std. err.) |
| Gender (base: man) |  |  |
| Woman | 0.12***  (0.02) | 0.05***  (0.03) |
| Field (mis)match  (base: match, higher-paid field) |  |  |
| Mismatch | 0.11***  (0.02) | 0.08***  (0.02) |
| Match, lower-paid field | 0.29***  (0.02) | 0.23***  (0.03) |
| Gender*field |  |  |
| Woman*Mismatch |  | 0.06*  (0.03) |
| Woman*Match, lower-paid field |  | 0.13**  (0.04) |
| Age | 0.01***  (0.00) | 0.01***  (0.00) |
| Country of birth (base: Germany) |  |  |
| Abroad | -0.01  (0.02) | -0.01  (0.02) |
| University type (base: university) |  |  |
| University of applied sciences | -0.01  (0.00) | -0.01  (0.00) |
| State (base: BB) |  |  |
| BE | -0.04  (0.04) | -0.04  (0.04) |
| BW | -0.01  (0.05) | -0.01  (0.05) |
| BY | -0.03  (0.05) | -0.03  (0.05) |
| HB | -0.05  (0.07) | -0.05  (0.07) |
| HE | -0.02  (0.04) | -0.02  (0.04) |
| HH | 0.04  (0.05) | 0.04  (0.05) |
| MV | -0.00  (0.06) | -0.00  (0.05) |
| NI | -0.02  (0.05) | -0.02  (0.05) |
| NW | -0.05  (0.05) | -0.05  (0.04) |
| RP | -0.00  (0.05) | -0.01  (0.05) |
| SA | 0.10  (0.12) | 0.10  (0.12) |
| SH | -0.07  (0.05) | -0.07  (0.05) |
| SL | -0.01  (0.05) | -0.02  (0.05) |
| SN | -0.01  (0.04) | -0.01  (0.04) |
| TH | -0.00  (0.06) | -0.00  (0.06) |
| Constant | -0.018** | -0.15* |
| N | 15,759 | 15,759 |
| R-squared | 0.074 | 0.076 |
| * p<0.05, ** p<0.01, *** p<0.001  Source: German Student Survey (Die Studierendenbefragung in Deutschland), 2021. | | |

**Table S3.** Linear probability models of unfairness perception, over different comparisons between fields of study.

|  | ***Eng. vs Hum.*** | ***Hum. vs Eco.*** | ***Hum. vs Law*** | ***Hum. vs Med.*** | ***Eng. vs Eco.*** | ***Eng. vs Law*** | ***Eng. vs Med.*** | ***Law vs Eco.*** | ***Med. vs Eco.*** | ***Med. vs Law*** |
| --- | --- | --- | --- | --- | --- | --- | --- | --- | --- | --- |
|  | Coefficient (robust std. err.) | Coefficient (robust std. err.) | Coefficient (robust std. err.) | Coefficient (robust std. err.) | Coefficient (robust std. err.) | Coefficient (robust std. err.) | Coefficient (robust std. err.) | Coefficient (robust std. err.) | Coefficient (robust std. err.) | Coefficient (robust std. err.) |
| Sex (base: man) |  |  |  |  |  |  |  |  |  |  |
| Woman | 0.10*  (0.05) | 0.22*** (0.04) | 0.09  (0.05) | 0.06  (0.03) | 0.17***  (0.04) | 0.17*** (0.03) | 0.10** (0.03) | 0.14*** (0.04) | 0.07** (0.03) | 0.06*  (0.03) |
| Field (mis)match  (base: match, higher-paid field) |  |  |  |  |  |  |  |  |  |  |
| Mismatch | 0.20* (0.09) | 0.17*** (0.04) | 0.21***  (0.06) | 0.09  (0.08) | 0.17** (0.06) | 0.13*** (0.04) | 0.15*  (0.06) | 0.17*** (0.04) | 0.06  (0.05) | 0.15***  (0.04) |
| Match, lower-paid field | 0.45***  (0.09) | 0.39*** (0.05) | 0.41***  (0.07) | 0.27** (0.08) | 0.14 (0.08) | 0.22*** (0.06) | 0.24*** (0.07) | 0.21*** (0.05) | 0.10  (0.07) | 0.32***  (0.08) |
| Constant | -0.66**  0.24 | 0.14  0.15 | -0.09  0.18 | -0.21  0.17 | -0.39**  0.13 | -0.13  0.15 | -0.01  0.18 | -0.09  0.18 | -0.18  0.13 | -0.17  0.10 |
| N | 1,561 | 1,582 | 1,549 | 1,680 | 1,556 | 1,575 | 1,540 | 1,557 | 1,557 | 1,602 |
| R-squared | 0.204 | 0.249 | 0.088 | 0.102 | 0.137 | 0.106 | 0.061 | 0.092 | 0.047 | 0.080 |

* p<0.05, ** p<0.01, *** p<0.001

Notes: All controls and weights included (not presented).

Source: German Student Survey (Die Studierendenbefragung in Deutschland), 2021.

**Section C: Robustness checks**


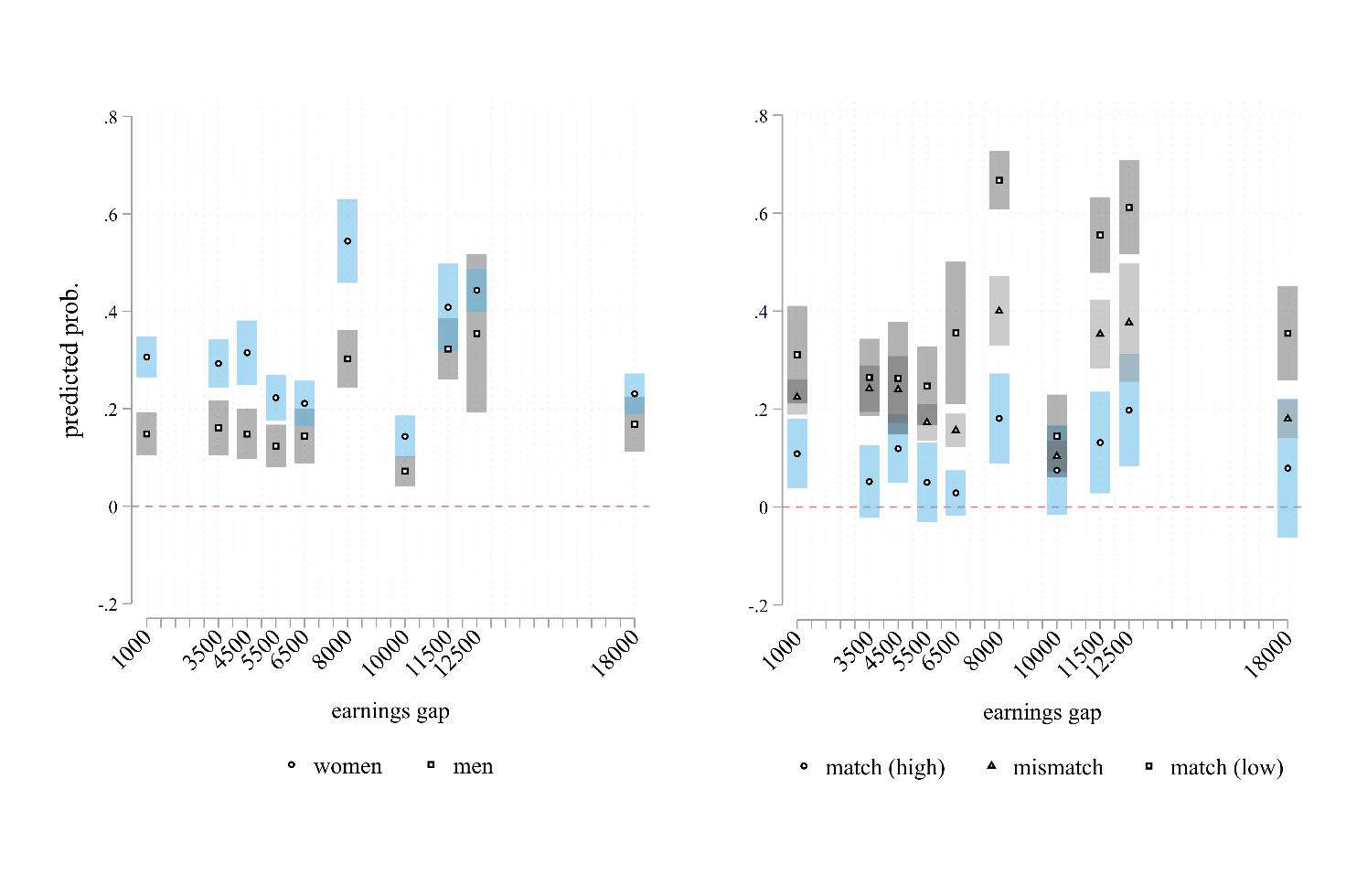


**Figure S1.** Predicted probabilities of perceiving unfairness by respondents’ gender (left panel) and the match or mismatch between respondents’ and experimental field of study (95% c.i.).

Notes: N: 15,759.

Source: German Student Survey (Die Studierendenbefragung in Deutschland), 2021.


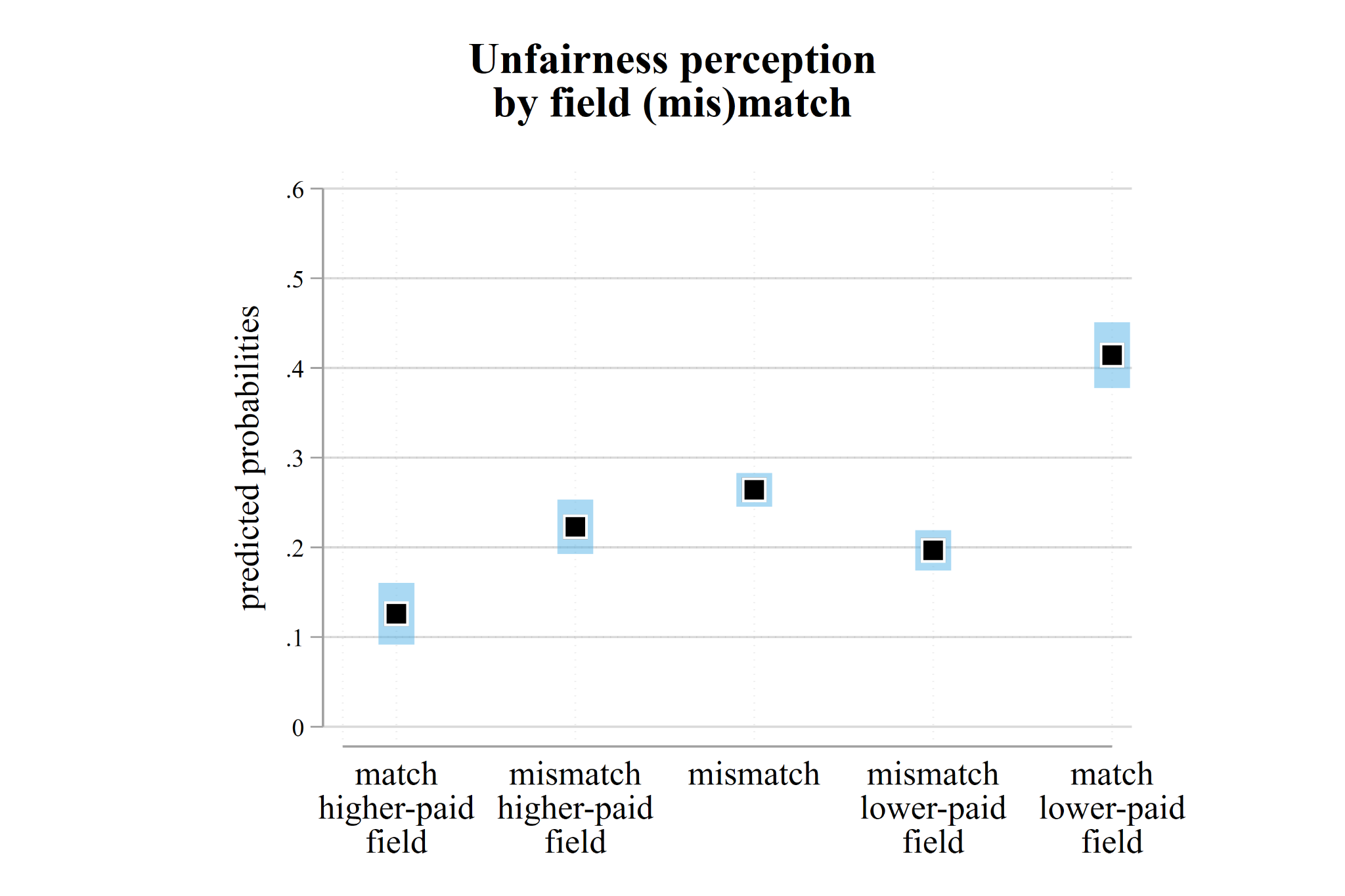


**Figure S2.** Predicted probabilities of perceiving unfairness by respondents’ match or mismatch between respondents’ and experimental field of study. Different categorisation of match/mismatch (95% c.i.).

Notes: N: 15,759.

Source: German Student Survey (Die Studierendenbefragung in Deutschland), 2021.


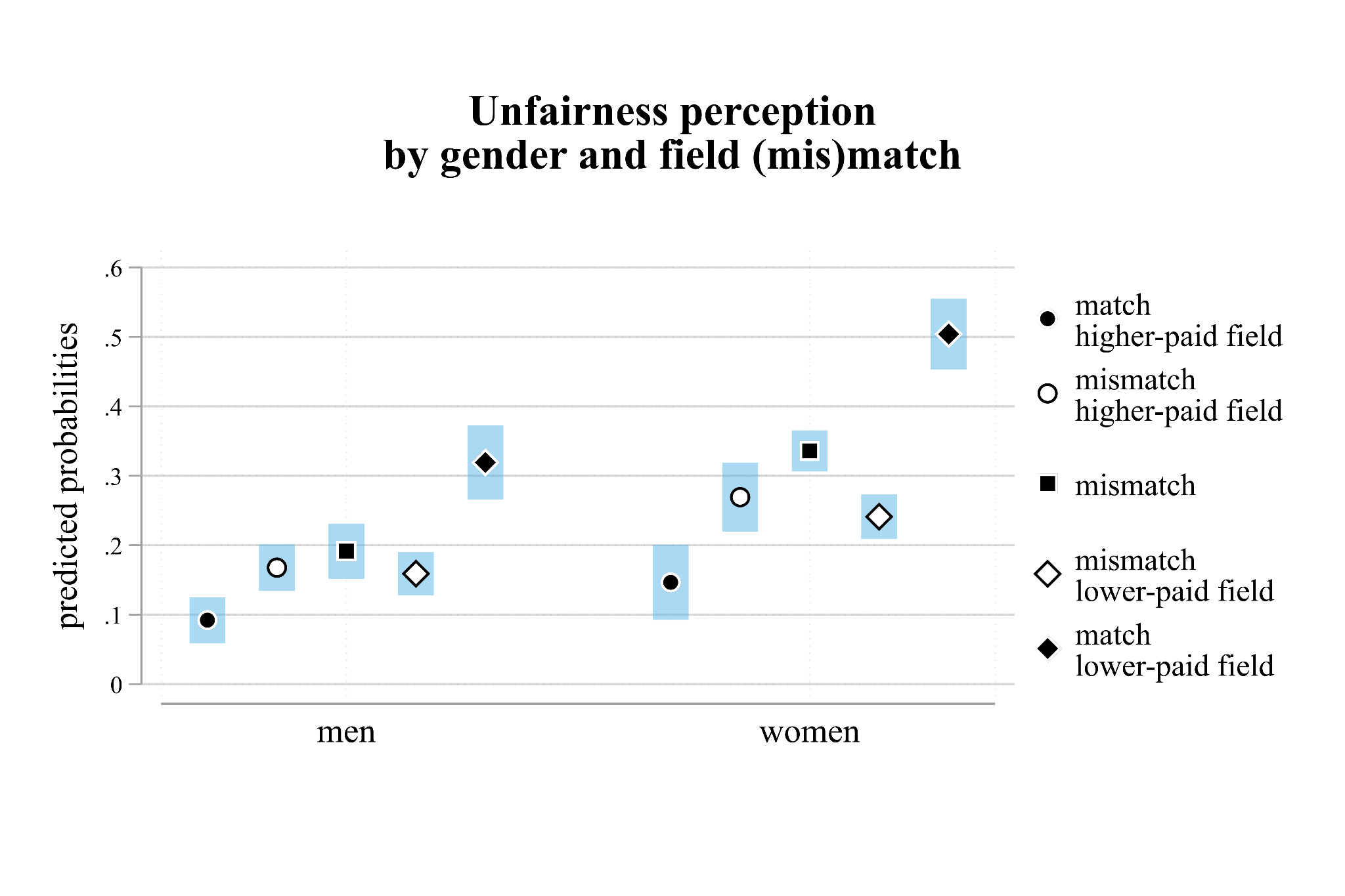


**Figure S3.** Predicted probabilities of perceiving unfairness by respondents’ gender and the match or mismatch between respondents’ and experimental field of study. Different categorisation of match/mismatch (95% c.i.).

Notes: N: 15,759.

Source: German Student Survey (Die Studierendenbefragung in Deutschland), 2021.

**Section D: Potential justifications of earnings differentials**

In this section, we conducted an exploratory analysis of the possible reasons for justifying the higher earnings in the comparison. The 11 reasons originally present in the experiment are outlined in section A. No upper or lower limits were applied, so that each respondent could select as many reasons as they wished. Table S4 shows our renaming of the original items and their grouping (useful for a more informative presentation of the results). We grouped these reasons into three distinct groups, which we identified as follows: (i) effort and responsibility; (ii) skill and economic relevance; and (iii) societal value.

**Table S4.** Reasons invoked to justify why graduates in one field of study are better paid than graduates in another field. Original survey items, their renaming, and grouping.

| **Groups of reasons** | **Reasons**  **(renamed)** | **Reasons**  **(original phrasing in the survey)** |
| --- | --- | --- |
|  |  | Individuals graduating from [field of study]... |
| Effort and responsibility | Study requirements | Had a higher level of requirements in their studies |
|  | Work intensity | Have a longer working week |
|  | Limited flexibility | Have less temporal/spatial flexibility in their work |
|  | Stress | Have more stress/strain at work |
|  | Complex tasks | Perform more complex tasks at work |
|  | Responsibility | Take on more responsibility at work |
|  |  | Individuals graduating from [field of study]... |
|  | On-demand skills | Have knowledge and skills that are more in demand on the labour market |
| Skill and economic relevance | National economy relevance | Have a job that is of greater importance to the economy |
|  | Financial returns | Generate high financial returns |
|  |  | Individuals graduating from [field of study]... |
| Societal value | Social relevance | Fulfil an important social function |
|  | Life-quality contributor | Contribute to securing the quality of life in society |

Source: German Student Survey (Die Studierendenbefragung in Deutschland), 2021.

In order to provide a more nuanced picture of the potential reasons for the fairness evaluations, the results are presented by comparisons between the fields evaluated by respondents, grouped according to the higher-paid field (engineering, law, economics, or medicine). As no significant differences were found between male and female respondents, or between students enrolled in different fields of study, we favoured the most parsimonious presentation of the overall results and do not report results by respondent characteristics. The coefficients in Figure S4 refer to the predicted probability of mentioning (compared to not mentioning) each justification. Weights accounting for the sampling strategy and population characteristics are applied. No control variables are included in the models.

Effort and responsibility in the educational and career path are among the most cited reasons for the higher earnings associated with medicine, followed by law. Higher levels of responsibility and stress are the items most commonly mentioned (with predicted probabilities reaching 0.7). Reasons related to skills that are in demand, and the economic relevance of the fields of study are instead more commonly invoked in comparisons involving economics and engineering. Being associated with skills that are in demand in the labour market is the item most often chosen to justify earnings differentials related to these disciplines (with predicted probabilities between 0.5 and 0.6). Finally, societal value factors are more likely to be mentioned in comparisons involving medicine. This is especially the case for the item regarding the important societal function fulfilled by graduates in this discipline, followed by their contribution to quality of life.


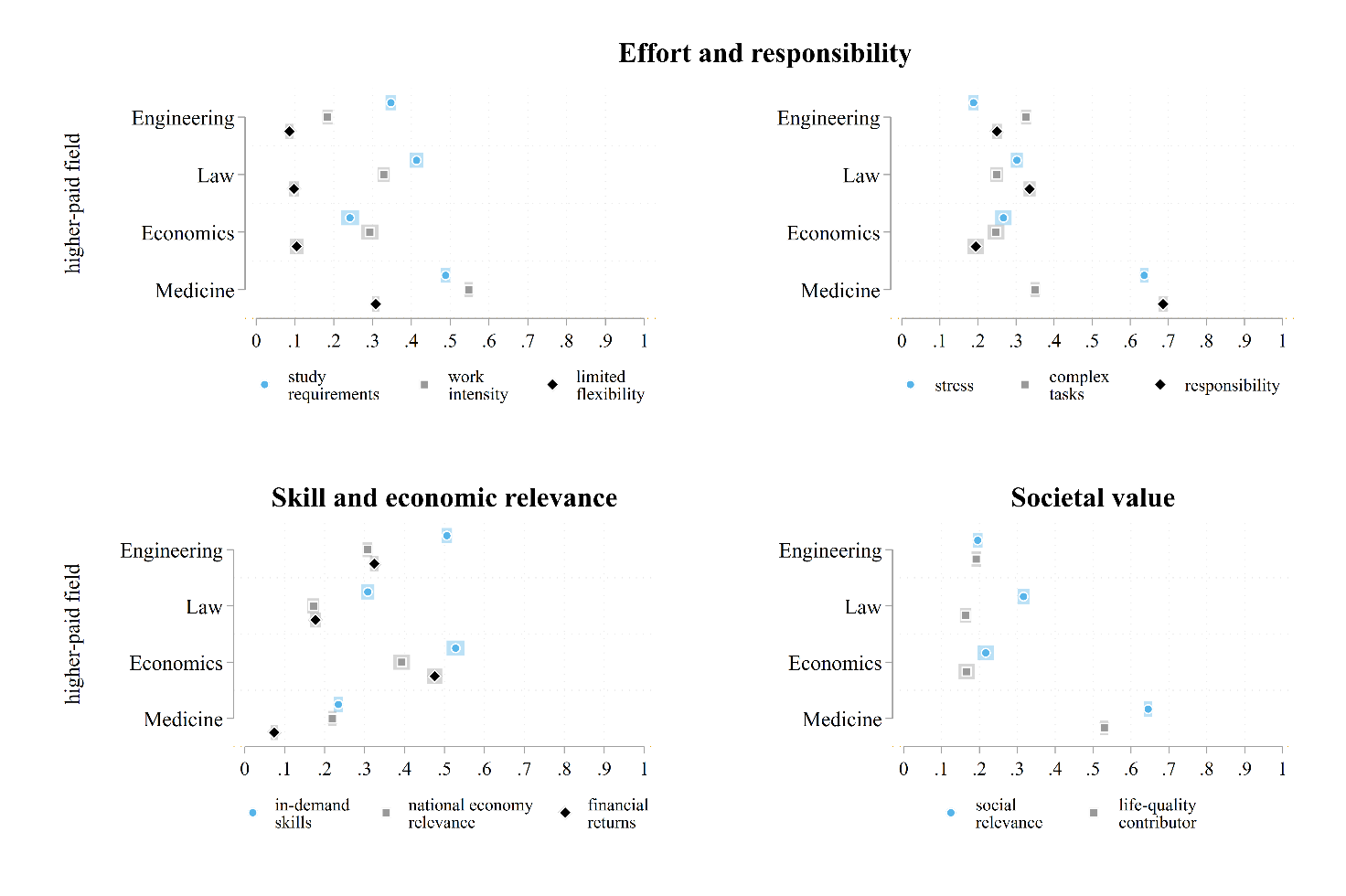


**Figure S4.** Predicted probabilities of mentioning different reasons to justify the higher wages in a given comparison (95% c.i.).

Notes: N: 15,759.

Source: German Student Survey (Die Studierendenbefragung in Deutschland), 2021.
